# The spatial pattern and influencing factors of tourism development in the Yellow River Basin of China

**DOI:** 10.1371/journal.pone.0242029

**Published:** 2020-11-18

**Authors:** Shengrui Zhang, Guanghai Zhang, Hongrun Ju

**Affiliations:** 1 Management College of Ocean University of China, Qingdao, China; 2 School of Tourism and Geography Science, Qingdao University, Qingdao, China; Institute for Advanced Sustainability Studies, GERMANY

## Abstract

With the implementation of the Belt and Road Initiative and the national strategy of “Ecological Protection and High-Quality Development in the Yellow River Basin”, the tourism development of the Yellow River basin of China is facing important opportunity. However, the spatial differences of tourism economy and the unbalanced development of interprovincial resources has become a threat for the sustainable development of the basin. By using the statistical data from 2003 to 2018, this paper aims to identify the numerical feature and spatial patterns of tourism development in the Yellow River Basin from the aspects of tourist volume (domestic tourists and inbound tourists) and tourism income (income from domestic tourism and inbound tourism) at provincial and prefectural scales. Analysis of spatial autocorrelation reveals significant clusters and outliers of tourist volume and tourism income at prefectural scale. Location condition, terrain condition, culture resources, regional policies, the interregional relationship and tourism infrastructure were the main factors influencing the spatial differences of tourism development in the Yellow River Basin. The study could offer useful information for the regional tourism management in the Yellow River Basin.

## Introduction

The Yellow River Basin was the center of Chinese politics, economy and culture for over 2000 years. There are numerous world-famous tourism attractions in the basin. Due to its long history, the Yellow River Basin owns many historical and cultural heritage, including the four great ancient capitals of China (the cities of Xi’an, Luoyang, Kaifeng and Anyang), the three grotto sites (Yungang Grottoes, Longmen Grottoes, Maijishan Grottoes) and the four great mountains (Mountain Tai, Mountain Hua, Mountain Song and Mountain Heng). Besides, there are diverse ethnic cultures in the basin compared with other areas in China. For example, the Buddhism in Qinghai and Inner Mongolia are much different than that in the other area [1]. In addition, there are a variety of natural landscapes distributed in the Yellow River Basin, for example: Yellow River Grand Canyon, Sanjiangyuan National Park, Gyaring Lake, Ngoring Lake, Ulansuhai Nur, Hukou Waterfall, Sand Lake and Shapotou National Nature Reserve.

In recent years, the harmonious development of tourism in the Yellow River Basin has been attached great importance. The Belt and Road Initiative launched by China in September 2013, clearly identified “tourism cooperation” as one of the key elements of "people-to-people connection" between China and the countries along the Belt and Road. As the starting area and important hinterland of the Silk Road, the development of the Yellow River Basin has attracted much attention. The Ecological Protection and High-quality Development of the Yellow River Basin was proposed as national strategy in September 2019, and the main objective of the strategy was to protect, inherit and promote of the Yellow River culture. In addition, the Yellow River Basin is an important region for winning the battle against poverty in China. By May 2019, 37.1% of the impoverished counties of China are located in the Yellow River Basin. The provinces of Qinghai, Sichuan, Gansu, Shaanxi, Ningxia and Inner Mongolia along the River are less developed compared with the eastern areas in China. The poverty in the junction areas of Gansu, Qinghai and Sichuan is even more serious due to the complex terrain which seriously blocked the transportation and the economic activities and the negligent management by government. However, these poor areas have rich and diverse natural tourism resources, which are ecologically friendly and authentic, indicating great potential for tourism development. In November 2016, a document named “the 13th Five-Year Plan for Poverty Alleviation” was published by the State Council aiming at eradicating poverty, and tourism was identified as one important measure to boost poverty area development. These positive policies indicate a booming exploitation of tourism development in the Yellow River Basin.

With the boom of the tourism industry, the understanding of tourism system develops at the same time. As a multi-structure system, a tourism system has extremely complex constituent structure, internal form and specific functions, and is in the process of constant change and development [2]. Based on the constituent elements and spatial model, the researchers analyzed the spatial structure of the tourism system and discussed the optimization measures for the balanced development of regional tourism [3]. One of the most concerned content of the tourism system is the development of interregional tourism. The interregional tourism resources have three features: spatial integrity, cultural homology and resource complementarity [4,5]. There are many influencing factors of interregional tourism development, including the characteristics of interregional tourism resources, the management policy and the traffic conditions [6–8]. In order to realize the harmonious development of interregional tourism, it is essential to discuss the competition and cooperation in the regional development [9–12]. Based on the scale of tourism economy and the strength of competition, there are three types of relationship among the neighboring administrative units in the development of interregional tourism: weak-weak, strong-weak and strong-strong [13,14].

The current research on the tourism development of the Yellow River Basin focused on the evaluation of tourism resources [15,16], the interregional cooperation [17], and the relationship between the tourism development and ecological protection [18,19]. However, the study areas are always limited in one provincial unit, while the study on the entire basin was rare. There are multiple administrative units in Yellow River Basin, and the harmonious development between different administrative units is the core issue of the basin management. Due to the administrative division, the tourism development in the Yellow River Basin is constantly unbalanced, and the development of interregional tourism is extremely unbalanced [17]. At basin level, the construction of the Yellow River Tourism Corridor should adopt the strategy of block integration and coordinated development [20]. At provincial level, the tourism industry was identified as one of the pillar industries in the Yellow River Basin. The interregional tourism cooperation is mature and representative in the junction area of Shanxi, Shaanxi and Henan, which is called golden triangle of the Yellow River. In addition, affected by the different policies in the different administrative units, the interregional tourism development is unbalanced and the abundance of tourism resources is inconsistent with the regional tourism development in the Yellow River Basin.

This paper intends to analyze and visualize the numerical features and spatial patterns of tourism development from the tourist volume and tourism income at provincial and prefectural level in the Yellow River Basin from 2003 to 2018. Then, the factors influencing the spatial pattern of the tourism development will be discussed. The results will be helpful for the tourism management in the Yellow River Basin and other interregional areas.

## Data and methods

### Study area

**Fig 1:**
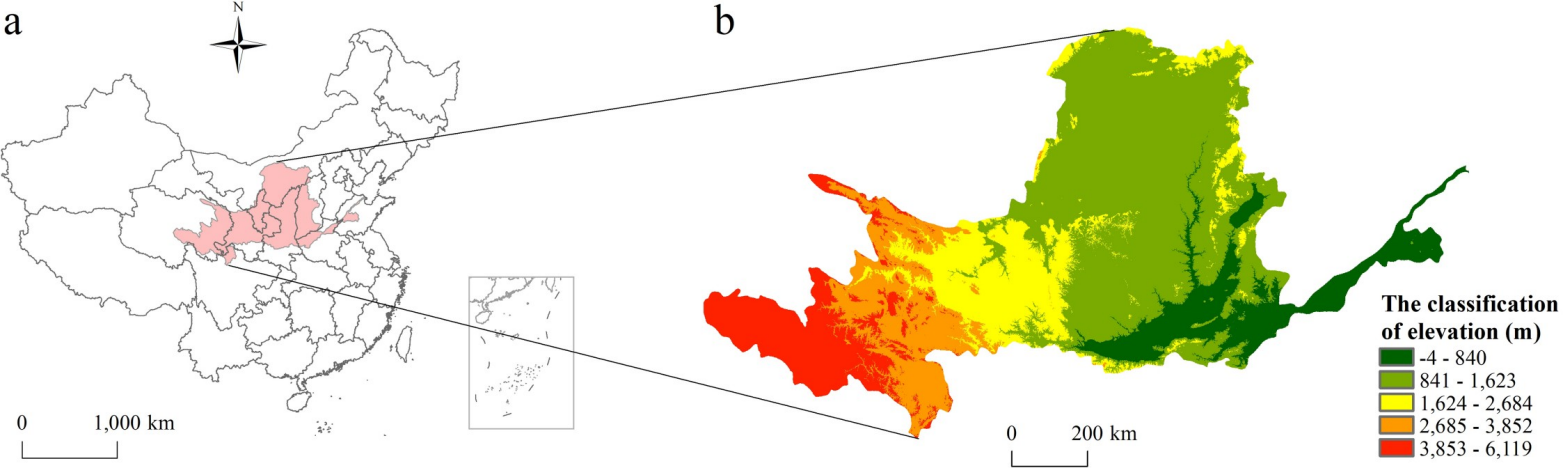
The Basic condition of the Yellow River Basin (a: Location of the study area; b: Topography of the study area).

The Yellow River Basin located in the central part of China (Fig 1A). Originating in the Bayan Har Mountains in Qinghai province of Western China, the Yellow River Basin empties into the gulf of Bohai Sea in Shandong province. The Yellow River Basin has an east-west extent of about 1900 km and a north-south extent of about 1100 km. Its total drainage area is about 795000 km^2^. The terrain of the Yellow River Basin fluctuates greatly, and the elevation of the basin gradually decreases from west to east (Fig 1B). The natural conditions such as topography, climate, hydrology and vegetation are complex. In addition, the social economic development of the Yellow River Basin is significantly different. The economy in the eastern parts of the basin is more developed than the western parts. Besides the Han culture, there are Tibetan culture, Muslim culture, Mongolian culture and other minority culture distributed in Yellow River Basin, and the differences of religion and folk custom of different culture is obvious.

**Fig 2:**
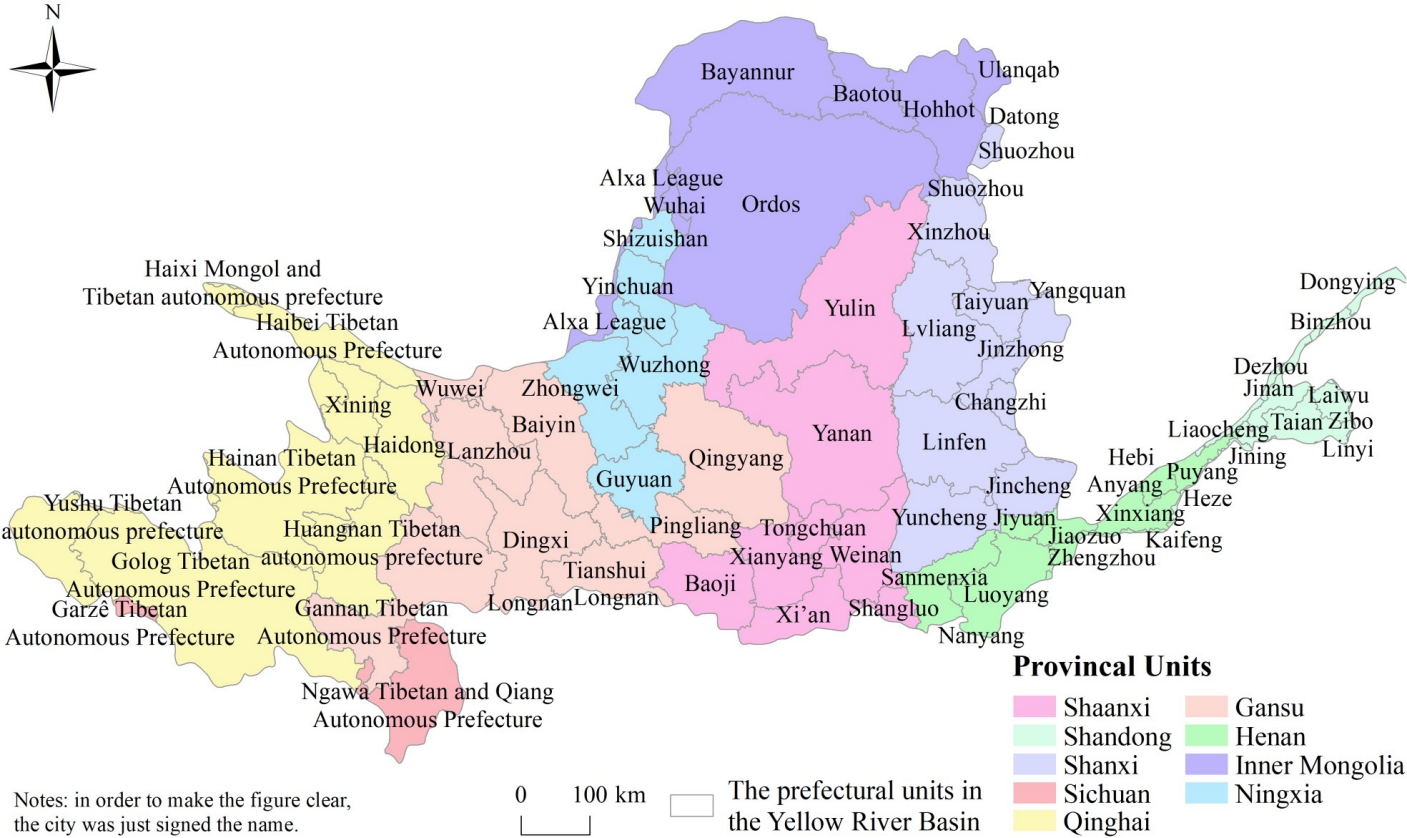
The prefectural units along Yellow River.

The Yellow River flows through 9 provinces, 73 prefectural units (62 cities, 10 autonomous prefectures and 1 league) (Fig 2). There are obvious differences in the number of prefectural units located in the Yellow River Basin of each province. The provincial units of Shanxi, Shandong and Henan, has 11 prefectural units in the basin, while Sichuan province only has 2 prefectural units in the basin (Fig 2).

### Data source

With reference to the classification of tourism economic indicators in the Yearbook of China Tourism Statistics 2003–2018 (http://tongji.cnki.net/overseas/brief/result.aspx), this paper selects two indicators to describe the development of tourism economy: the number of tourists and tourism income. The economic data of tourism were acquired from the statistical yearbook of each prefecture. In order to avoid the statistical differences caused by the adjustment of administrative divisions, this paper adopts the data of administrative divisions in 2018 (http://www.resdc.cn/data.aspx?DATAID=201). The data of DEM is in raster format with a 500-m resolution, provided by the National Fundamental Geographical Information System of China.

### Spatial pattern indicators

This paper analyzes the spatial pattern of tourism development in the Yellow River basin from two aspects: spatial variation and spatial association. The coefficient of variation (*CV)* was used to describe the spatial variation. The global and local Moran’s *I* indexes were used to describe the global and local spatial association of tourist volume or tourism income. And, the Getis-Ord General G was used to detect the hot and cold spot of the tourist volume and tourism income.

#### Spatial variation indicator

The *CV* was selected to measure the spatial variation of tourism economy at prefectural scale. It was often expressed as a percentage, and was defined as the ratio of the standard deviation (*SD*) to the mean (Eqs 1 and 2) [21,22]. The coefficient of variation (*CV*) was calculated as follows:

$$SD=\sqrt{\frac{1}{n}\sum_{i=1}^{n}{\left(X_{i}–\bar{X}\right)}^{2}}$$ (1)

$$CV=SD/\bar{X}$$ (2)

where the *CV* is the coefficient of variation; *SD* is the standard deviation; *n* is the number of divisions at a given scale (*n* = 73 at prefectural scale); *X_i_* is the tourists volume or tourism income of the *i*th division at a given scale (*i* = 1, 2, …, *n*.); and $\bar{X}$ is the mean number of the tourist volume or tourism income of the divisions. The larger the *CV* value, the more spatial variation in the tourism development.

#### Spatial association indicators

(1) Global and local indicators of spatial association

Spatial association statistics are used to measure and analyze the degree of dependency among divisions in a geographic space. In this study, the global Moran’s *I* and local Moran’s *I* were used to analyze the global and local spatial association of tourism economy, respectively.

$$\mathrm{Global}\;\mathrm{Moran}’s\;I=\frac{n}{\sum_{i=1}^{n}\sum_{j=1}^{n}w_{ij}}\frac{\sum_{i=1}^{n}\sum_{j=1}^{n}w_{ij}\left(X_{i}-\bar{X}\right)\left(X_{j}-\bar{X}\right)}{\sum_{i=1}^{n}{\left(X_{i}-\bar{X}\right)}^{2}}$$ (3)

where *n* is the number of spatial divisions; *X_i_* and *X_j_* are the tourists volume or tourism income in division *i* and *j* (*i*≠*j*); $\bar{X}$ is the average value; and *w_ij_* is the matrix of spatial weights. The values of the global Moran’s *I* range from −1 to +1. Positive values indicate the clustering of similar values across geographic space. In contrast, negative values indicate that the neighboring values are more dissimilar than expected by chance [23].

Local indicators of spatial association (LISA) could locate clustered patterns by comparing the tourist volume or tourism income in each specific location with the value in neighboring locations. The high-high clusters and low-low clusters were positive spatial associations of the tourist volume or tourism income. In contrast, spatial outliers were identified when a division with high tourist volume or tourism income was surrounded by neighboring divisions with low tourist volume or tourism income and vice versa [24].

$$Local\;Moran’s\;I=\frac{X_{i}-\bar{X}}{S_{i}^{2}}\overset{n}{\underset{j=1,j\neq i}{\sum }}w_{ij}(X_{j}-\bar{X})$$ (4)

where $S_{i}^{2}$ is the variance of the tourists’ number or tourism income, and the other symbols are the same as described above. A positive local Moran’s *I* value means a division has similar high or low values as its neighbors, while a negative local Moran’s *I* value means the location is significantly different from the surrounding areas [25,26].

(2) Getis-Ord General *G*

Global measures of spatial association analyze patterns on a large scale to show whether data are clustered, dispersed, or randomly distributed in space. As the global measures of spatial association can be used to test general patterns in data, the identification of statistically significant patterns of high (hot spots) or low (cold spots) attribute values within the study area is also interesting and necessary. One of the principal global indicators of association is the Getis-Ord General *G*. It estimates the overall level of spatial association for a dataset [27]:

$$G=\frac{\sum_{i=1}^{n}\sum_{j=1}^{n}w_{ij}x_{i}x_{j}}{\sum_{i=1}^{n}\sum_{j=1}^{n}x_{i}x_{j}},\forall j\neq i$$ (5)

where *x*_*i*_ and *x*_*j*_ are the tourist volume or tourism income for prefecture *i* and j, and *w*_*ij*_ is the spatial weight between prefectures *i* and *j*. *n* is the number of prefectures in the dataset and ∀ *j* ≠ *i* indicates that prefecture *i* and *j* cannot be the same one.

## Results

### The overall tourism development in the Yellow River Basin

**Fig 3:**
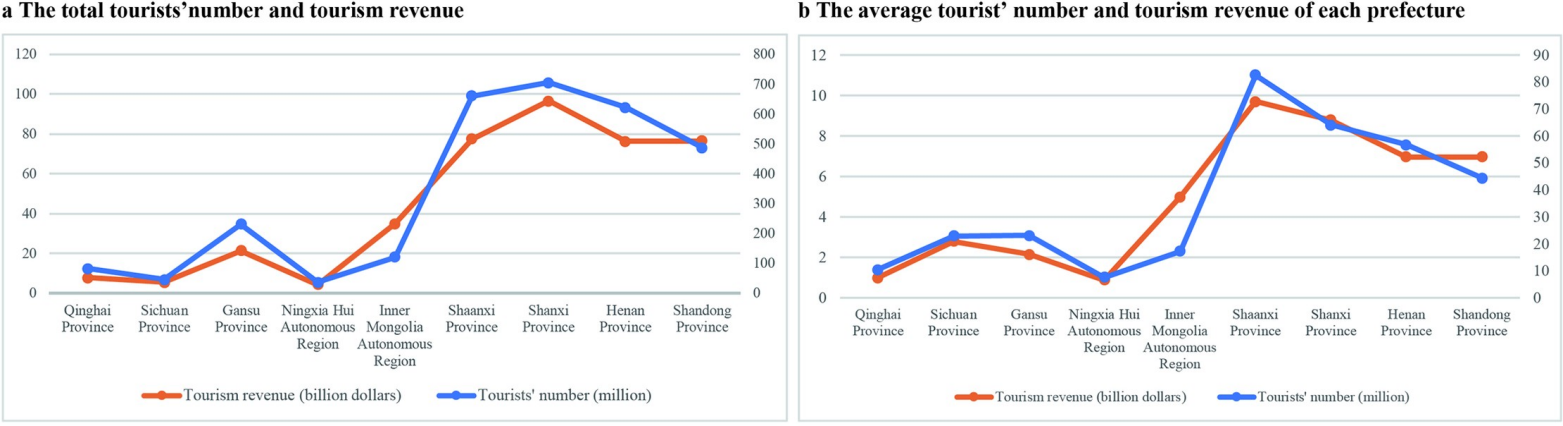
The development of tourism economy in the Yellow River Basin at provincial level.

At provincial level, Shanxi Province received the largest number of tourists (705.46 million) and the largest tourism income (96.60 billion dollars) in 2018, while Ningxia Hui Autonomous Region received the least tourists (37.77 million) and the least tourism income (4.42 billion dollars, Fig 3A). However, it is worth noting that the statistics was made for the part of each province located in the Yellow River Basin. The number of prefectures and the area that located in the Yellow River Basin of each province is different (Fig 2). Therefore, it is necessary to analyze the average tourists’ number and tourism income at prefectural level. It was found that the prefectures in Shaanxi Province had the largest number of the average tourists’ number (82.56 million per prefectural unit) in 2018, while the prefectures in Ningxia had the least number (7.55 million per prefectural unit). In the view of average tourism income at prefectural level, Shaanxi Province also had the largest number (9.70 billion dollars), while Ningxia had the least number (0.88 billion dollars, Fig 3B).

**Fig 4:**
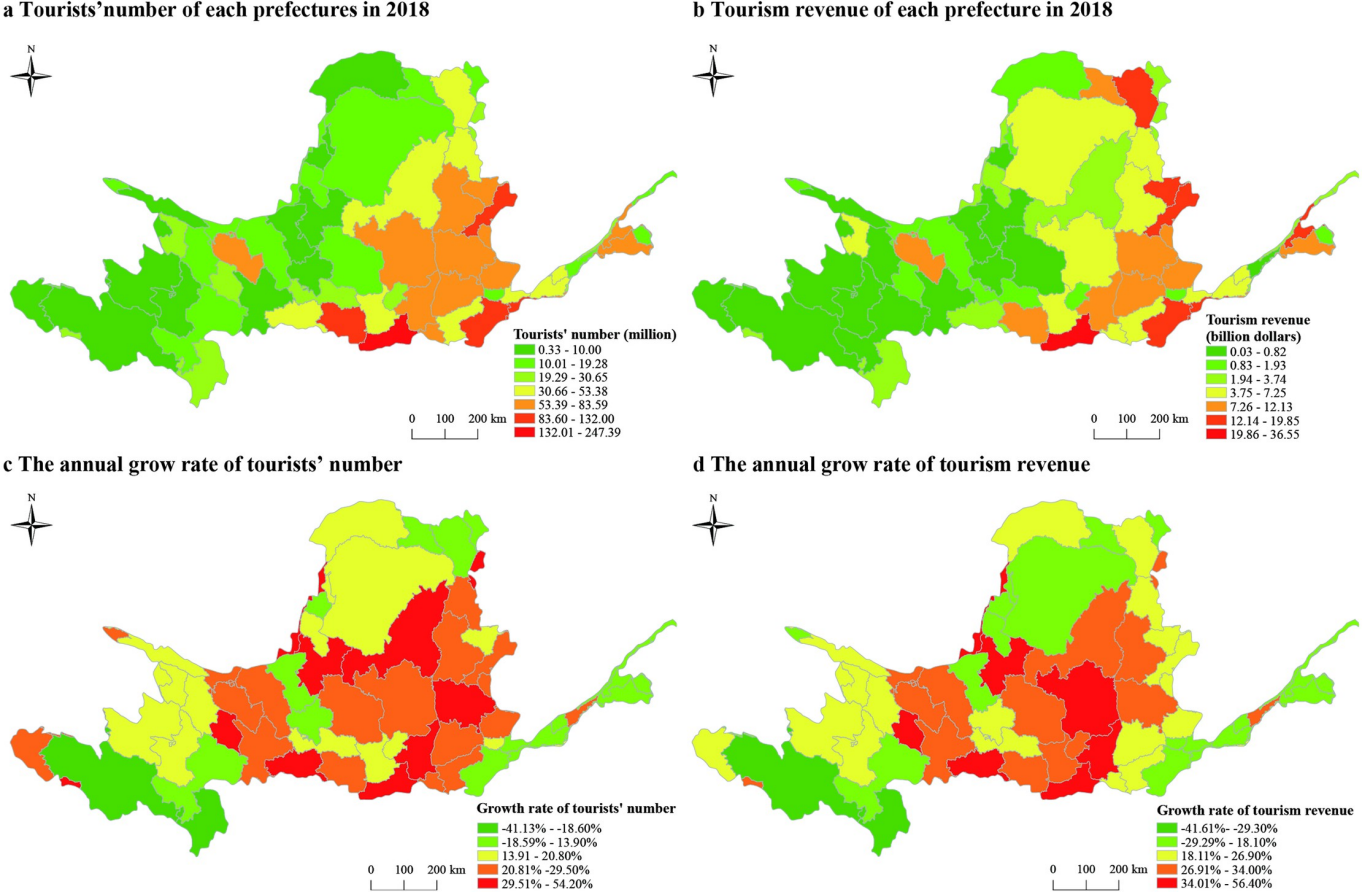
The numerical features of tourism economy in the Yellow River Basin.

At prefectural level, Xi’an city hosted 247.39 million tourists which is the largest one in the prefectural level. Golog Tibetan Autonomous Prefecture has the least number of tourists, with only 326000 (Fig 4A). In 2018, Xi’an city had the highest tourism income of 36.45 billion dollars, while Golog Tibetan Autonomous Prefecture had the lowest of 33.53 million dollars (Fig 4B).

During 2003–2018, there is an obvious increase in the tourists’ number of all prefectural units in the Yellow River Basin except Golog Tibetan Autonomous Prefecture and Ngawa Tibetan and Qiang Autonomous Prefecture. Overall, the annual growth rate is generally higher in the eastern prefectures than the western ones. The prefectures with the average annual growth rate higher than 35% are mainly distributed in the Inner Mongolia, Shaanxi and Gansu (Fig 4C). The average annual growth rate of Alxa League is the highest (54.20%), and other prefectures with more than 35% average annual growth rate including Weinan City (37.60%), Xi’an City (36.70%) and Tianshui City (35%). Only two prefectural units show a negative growth, which are Ngawa Tibetan and Qiang Autonomous Prefecture (-18.60%) and Golog Tibetan Autonomous Prefecture (-41.13%).

Compared with the tourists’ number, the growth of tourism income is more significant. The fast-growing provinces are Inner Mongolia, Shaanxi and Gansu. The fast-growing prefecture is Xi’an City (56.40%, Fig 4D), and the prefectures with the growth rate of tourism income over 35% including Alxa League (39.00%), Yan’an City (37.50%), Linxia Hui Autonomous Prefecture (37.03%), Wuzhong City (36.50%), Tianshui City (36.00%) and Weinan City (35.9%). The three prefectures with negative growth are Shizuishan City (-5.10%), Ngawa Tibetan and Qiang Autonomous Prefecture (-29.3%) and Golog Tibetan Autonomous Prefecture (-41.61%).

The results show that the prefectures with better development in the Yellow River Basin are concentrated in the eastern and central provinces, while the prefectures with rapid development of tourism are mainly concentrated in the central regions (Fig 4).

### The features of the domestic tourism in the Yellow River Basin

**Fig 5:**
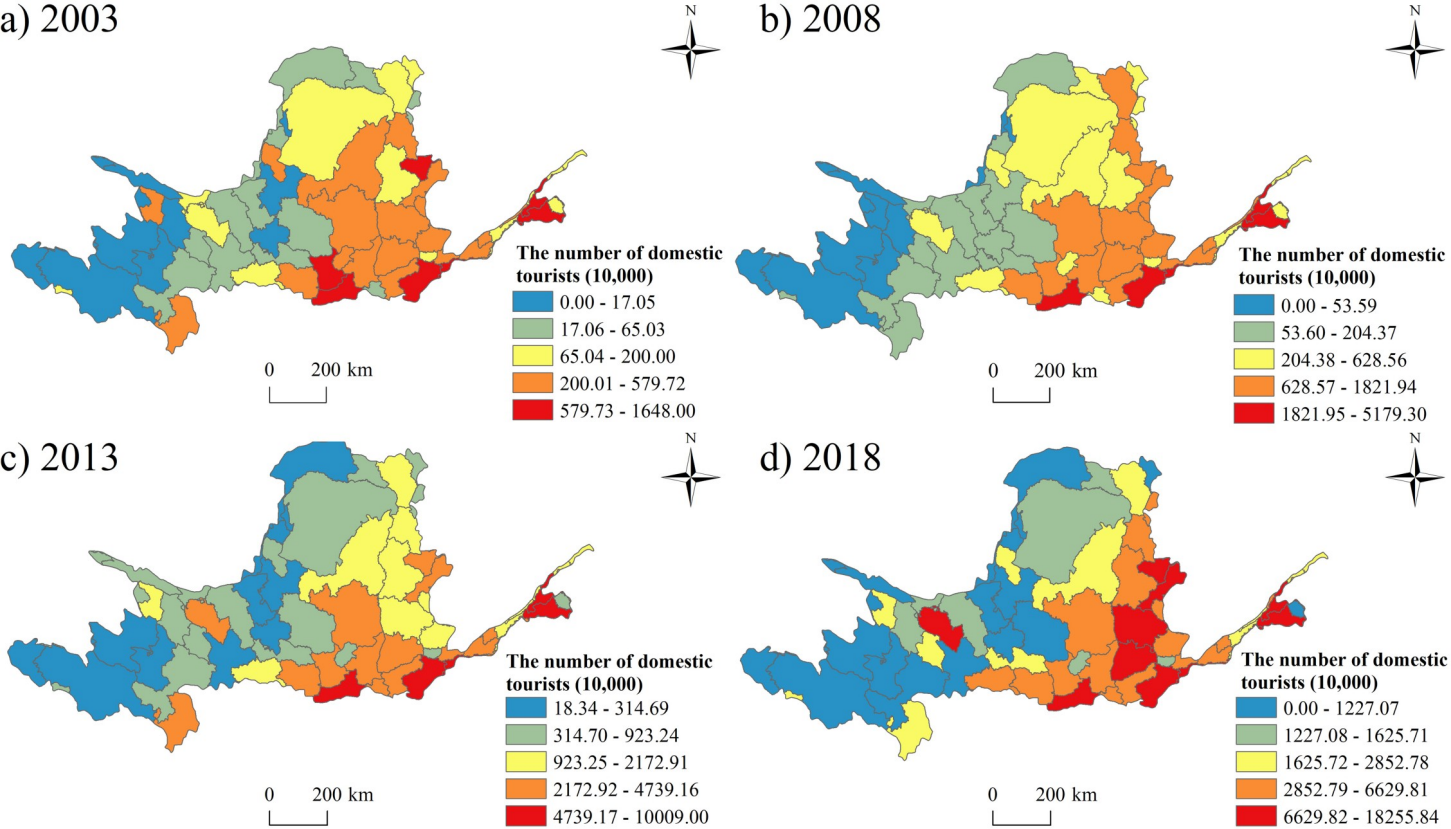
The spatial pattern of the domestic tourists’ number from 2003 to 2018.

According to the results of cross-sectional analysis, the domestic tourists increased obviously in the basin area, while the development gap between the east and west are growing gradually (Fig 5). Xi’an City in Shaanxi Province, Zhengzhou City and Luoyang City in Henan Province, Taiyuan City, Jinzhong City and Yuncheng City of Shanxi Province, Jinan City and Taian City of Shandong Province are always the hotspots in the domestic development. These cities are all historical city with rich cultural tourism resources. Xi’an, Zhengzhou, Taiyuan and Jinan are the provincial capital cities, which has convenient transportation facilities.

**Fig 6:**
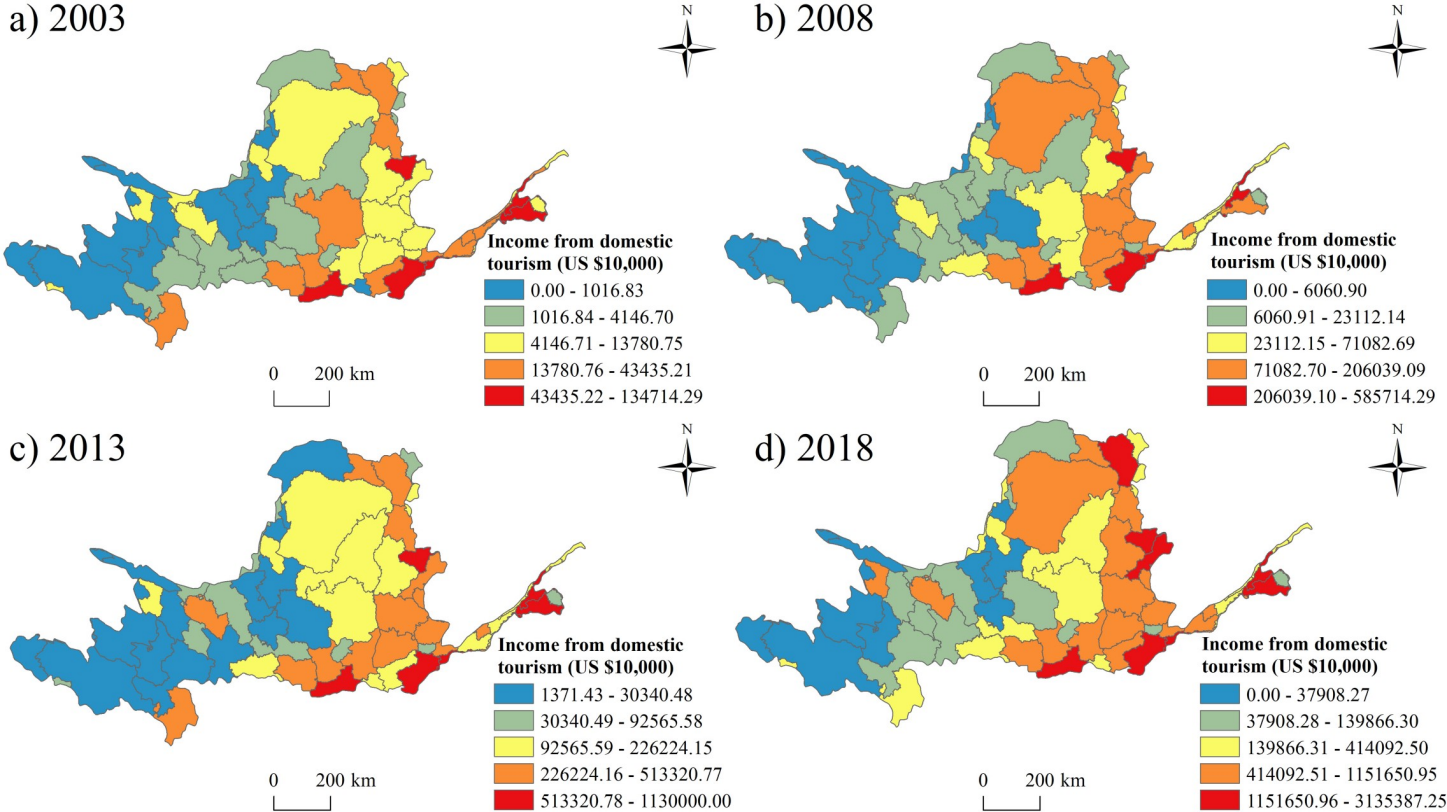
The spatial pattern of the domestic tourism income from 2003 to 2018.

The spatial pattern of domestic tourism income is similar with the tourists’ number, which also showed that the income from domestic tourism in the east province is higher than the west province (Fig 6). The hotspots were Zhengzhou City, Luoyang City, Xi’an City, Jinan City, Jinzhong City, Taiyuan City, Hohhot City and Taian City.

### The features of the inbound tourism in the Yellow River Basin

**Fig 7:**
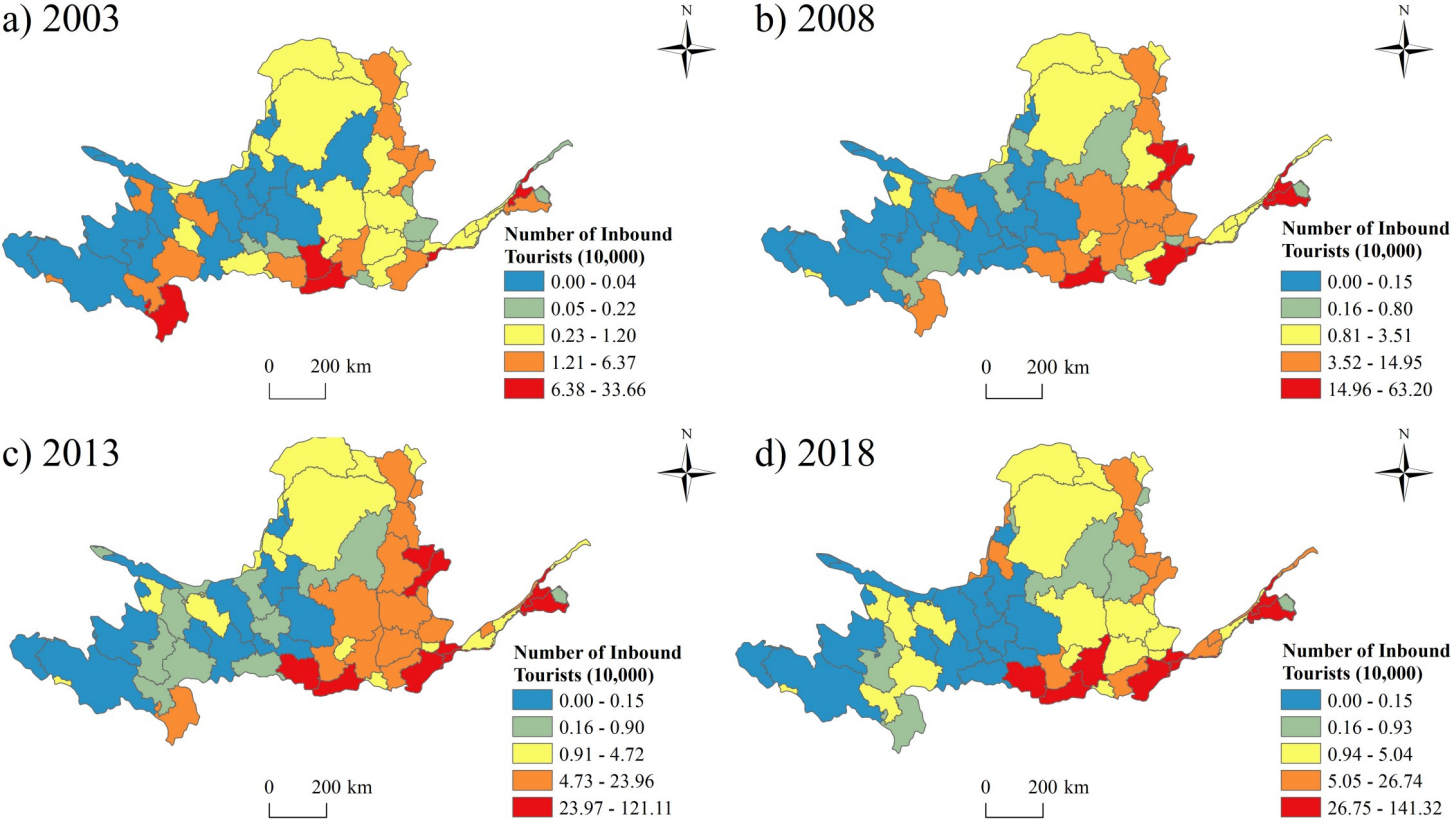
The spatial pattern of the number of inbound tourists from 2003 to 2018.

The inbound tourists increased quickly from 2003 to 2018, while the gap in the development between the east and west were growing larger (Fig 7). The largest number occurred in Luoyang City, which is famous for the only female emperor (Wu Zetian) and numerous grottoes.

**Fig 8:**
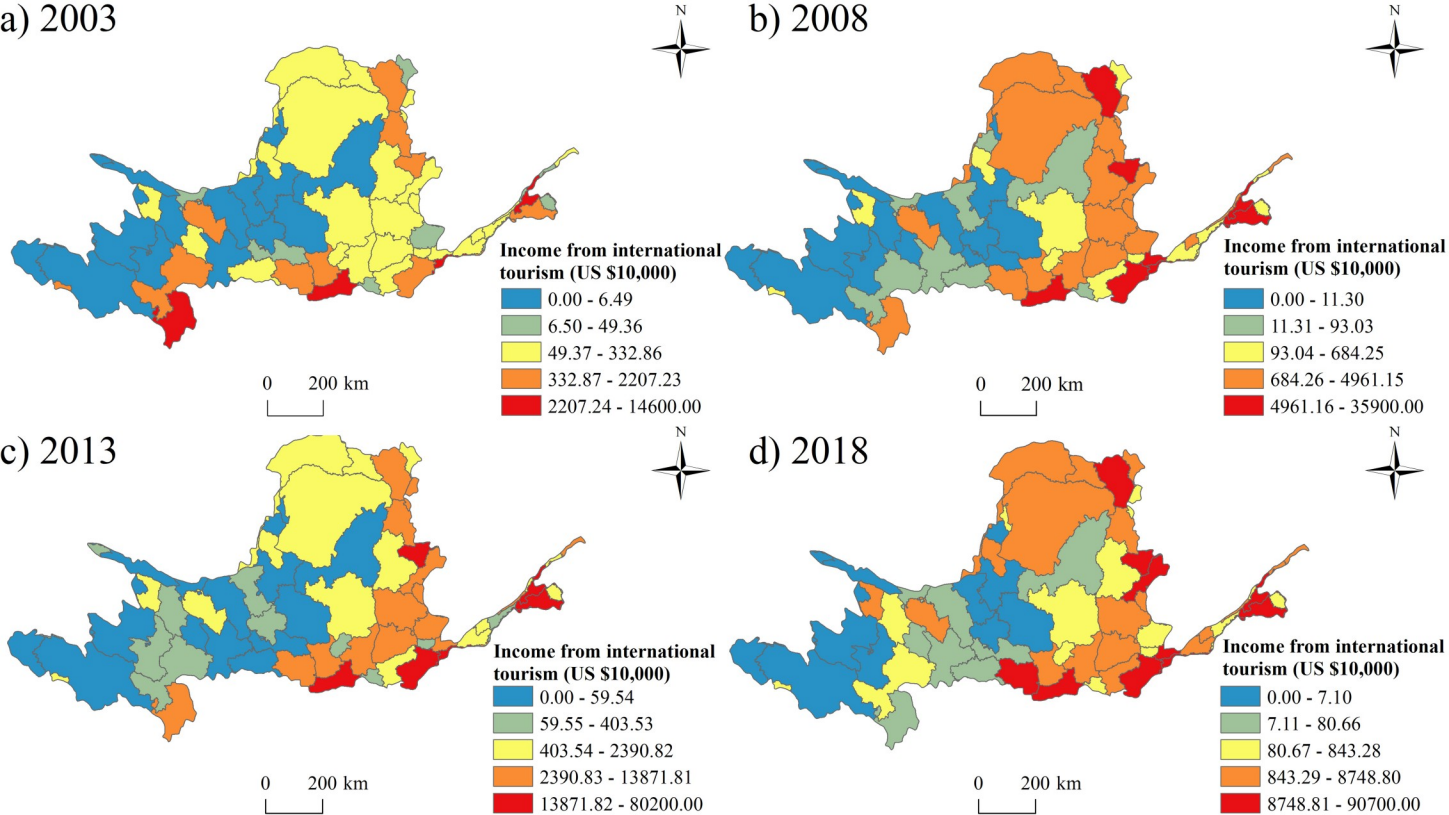
The spatial pattern of the inbound tourism income from 2003 to 2018.

There were obvious differences in the income from inbound tourism between the west and the east (Fig 8). The hotspots appeared in Xi’an City of Shaanxi province, which was famous for the ancient capital of China and the mountain Hua.

### Spatial variation of tourism development in the Yellow River Basin

The spatial variation of tourists’ number and tourism income was different at prefectural scale. The *CV* of tourists’ number and tourism income was 93.82% and 107.68%, indicating that the spatial distribution of the tourism income was more uneven than that of tourists’ number (Table 1).

**Table 1:** *CV*, Moran’s *I* and General *G* of tourist’s number and tourism income at different scales.

Indicator	tourists' number	tourism income
***CV***	93.82%	107.68%
**Moran *I***	0.1872**	0.1506**
**Expected General *G***	0.0139	0.0139
**Observed General *G***	0.0168**	0.0165**

Notes:

** indicates the value is significant at the 0.01 level.

**Fig 9:**
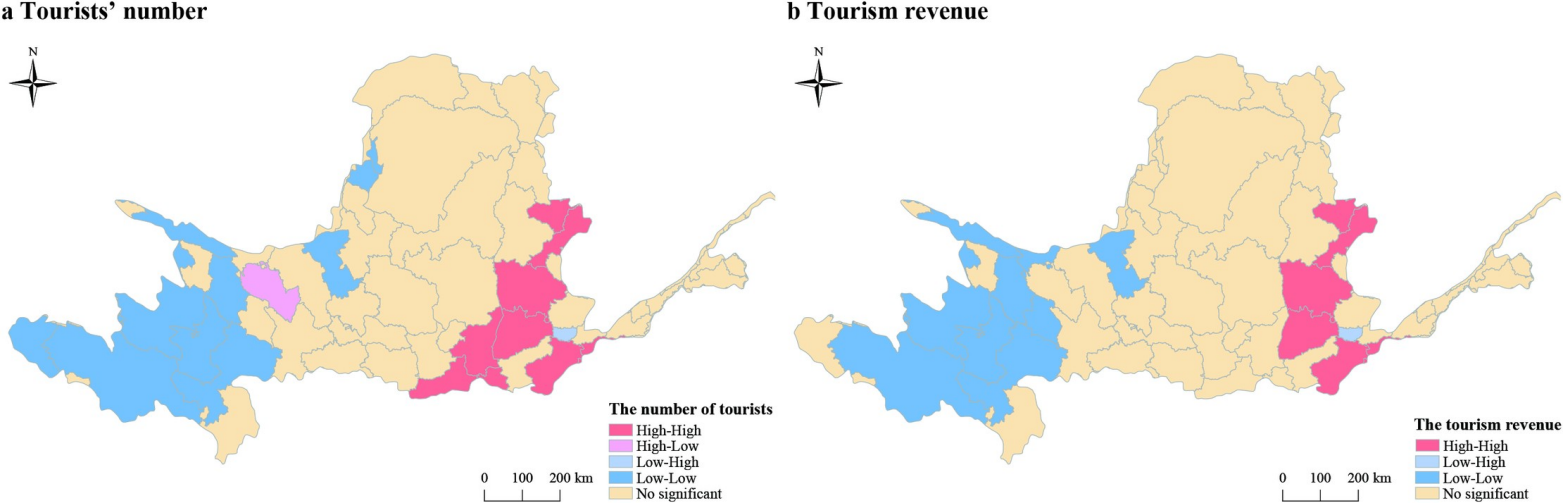
Local indicators of spatial association (LISA) map of tourists’ number and tourism income in the Yellow River Basin at prefectural scale.

The global Moran’s *I* of tourist arrivals and tourist income were all positively significant at 0.01 level (Table 1). It meant that the spatial agglomeration phenomenon of the tourist arrivals and tourist income was positively clustered at prefectural scale (Fig 9). The Observed General *G* was higher than the Expected General G in the tourists’ number and tourism income (Table 1). It meant that the spatial hot or cold area of the tourism economy was significant over the basin.

### Spatial clusters of tourism development in the Yellow River Basin

In terms of the tourists’ number, a cluster of prefectural units with more tourists, and neighboring prefectural units with more tourists, was apparent in the central part of Yellow River Basin. The prefectures of Taiyuan, Yangquan, Jinzhong, Linfen and Yuncheng in Shanxi Province, Weinan, Xi’an and Shangluo in Shaanxi Province, Luoyang and Zhengzhou in Henan Province even showed a significant cluster effect at 0.01 level. By contrast, clusters of prefectural units with less tourists, surrounded by neighbors with less tourists, was apparent in the west of Yellow River Basin including the prefectural units of Haixi Mongol and Tibetan autonomous prefecture, Yushu Tibetan Autonomous Prefecture, Golog Tibetan Autonomous Prefecture, Gannan Tibetan Autonomous Prefecture, Hainan Tibetan Autonomous Prefecture, Haidong City, Haibei Tibetan Autonomous Prefecture, Zhongwei City, Shizuishan City and Wuhai City. Lanzhou City in Gansu was the only significant high-low outlier, while Jiyuan City was the only significant low-high outlier (Fig 9A).

In terms of the tourism income, a cluster of prefectural units with higher revenue, and neighboring prefectural units with higher revenue, was apparent in the central part of the Yellow River Basin. The prefectures of Taiyuan, Yangquan, Jinzhong, Linfen and Yuncheng in Shanxi Province, Luoyang and Zhengzhou in Henan province even showed a significant cluster effect at 0.01 level. By contrast, clusters of prefectural units with lower revenue, surrounded by neighbors with lower revenue, was apparent in the western part of Yellow River Basin, including Golog Tibetan Autonomous Prefecture, Huangnan Tibetan Autonomous Prefecture, Hainan Tibetan Autonomous Prefecture, Haidong City and Haibei Tibetan Autonomous Prefecture in Qinghai, Gannan Tibetan Autonomous Prefecture, Linxia Hui Autonomous Prefecture, Wuwei City in Gansu and Zhongwei City in Ningxia. Jiyuan City in Henan was the only significant low-high outlier (Fig 9B).

It can be concluded that the spatial pattern of tourist arrivals and tourist revenue of the Yellow River Basin is similar, with high-high clusters in the middle and east, while low-low clusters in the west. It indicates that the tourism industry in the western region is generally weak and still has a large space for development.

### Spatial hot or cold area of tourism development in the Yellow River Basin

**Fig 10:**
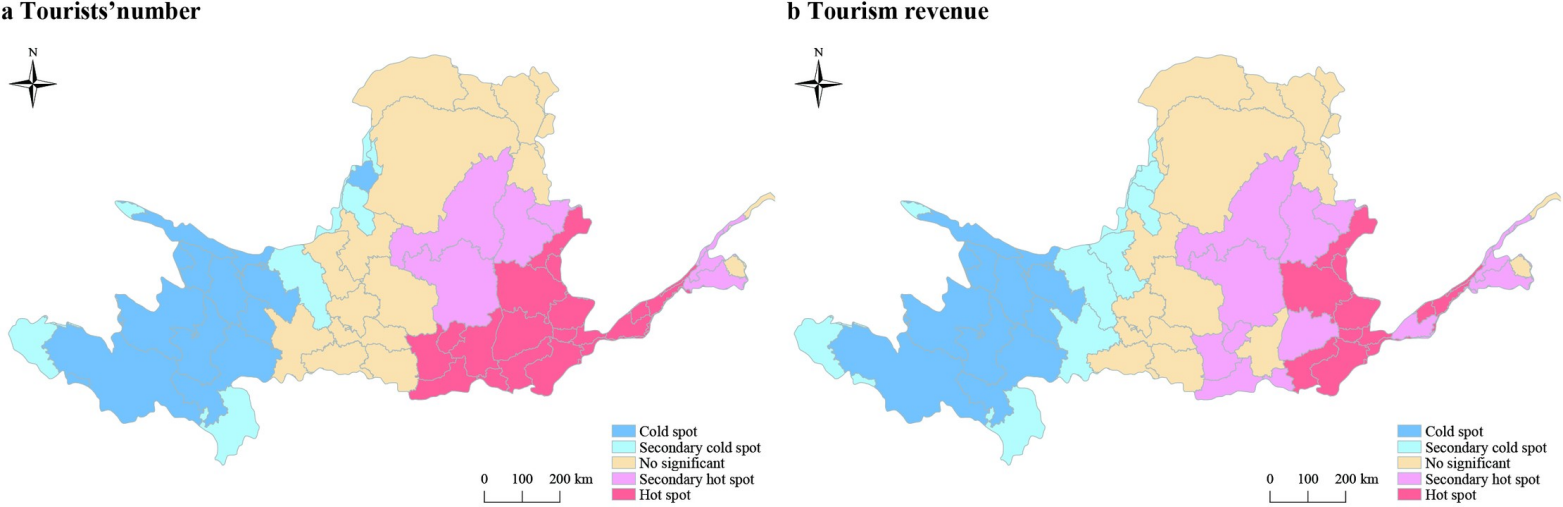
Hot/cold spots maps of tourism economy in Yellow River Basin at prefectural scale.

The hot spots of tourist arrivals were apparent in central and eastern part of Yellow River Basin. Provinces of Shandong and Henan, central and south of Shaanxi showed a significant cluster effect at 1% level. It indicates that the tourists gather in the lower reaches of Yellow River. By contrast, cold spots of tourist arrivals were clustered in western part of the basin. The prefectures in the provinces of Qinghai, Sichuan, Gansu and Ningxia showed a significant cluster effect at 1% level (Fig 10A). It indicates that the tourists rarely go to the upper reaches of the Yellow River.

In the case of tourism income, hot spots were gathered in eastern provinces of the Yellow River Basin, and the prefectures in the provinces of Henan, Shandong showed a significant effect at 1% level. There were two hot spots at the prefectural level, which is the clusters of the city of Zhengzhou, Jiaozuo, Luoyang and Sanmenxia, the other clusters of the city of Liaocheng, Puyang, Anyang and Hebi. Cold spots were gathered in west of the Yellow River Basin. Golog Tibetan Autonomous Prefecture, Yushu Tibetan Autonomous Prefecture and Huangnan Tibetan Autonomous Prefecture were the cold spots (Fig 10B).

According to the analysis of the hot and cold spots, the central and eastern parts are the hot spots of the Yellow River Basin tourism economy, while the western provinces are the cold spots.

## Discussion: The influencing factors of the tourism development in the Yellow River Basin

**Fig 11:**
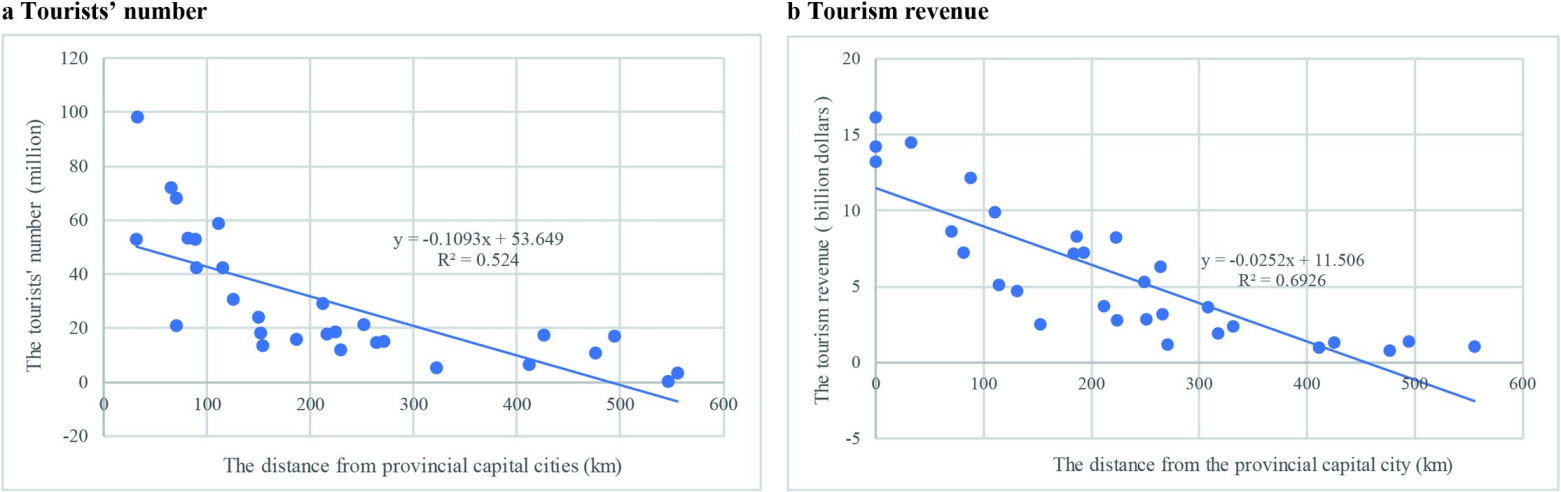
The linear relationship between the tourists’ number and the distances from provincial cities.

The location was an important factor for tourism development in the Yellow River Basin. By analyzing the relationship between the tourists’ number or tourism income and the distances from the provincial capital city (Fig 11), it can be seen that the farther the distance between the cities and the capital city is, the lower the tourists’ number or tourism income. Due to the complex terrain and imperfect transportation system, the travel cost will be higher in the western provinces. In addition, the domestic tourism markets in the western provinces are weaker. For example, the domestic tourism markets in the provinces of Qinghai and Gansu are not as developed as those of Shandong and Henan which had a larger population and located closer to Beijing-Tianjin megacity cluster.

**Fig 12:**
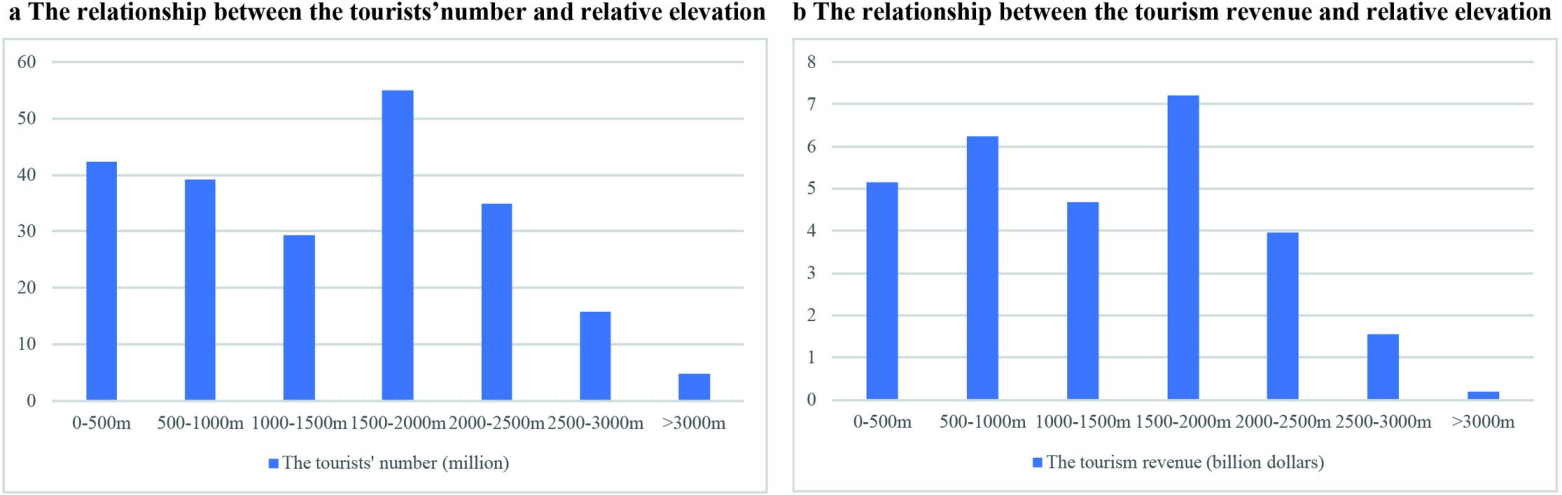
The relationship between the tourists’ number, tourism income and the relative elevation in Yellow River Basin.

Terrain condition exerted a significant influence on the spatial pattern of tourism economy in the Yellow River Basin. The statistic of the tourists’ number in different levels of relative elevation of the prefectural units showed that the tourists’ number was least in the prefectures with relative elevation above 3000m. It was because the complex terrain was a challenge for tourists to arrive. The largest number occurred in the relative elevation between 1500-2000m where the terrain was not so tough and the physical tourism resources were more diverse than the flat areas. When the relative elevation was above 2000 meters, there was a negative relationship between the tourists’ number and the relative elevation (Fig 12A).The relationship between the tourism income and the terrain showed a similar trend: the highest number occurred in prefectures with the relative elevation between 1500-2000m, and there was negative relationship between the tourism income and the relative elevation when the relative elevation more than 2000m (Fig 12B).

The cultural resources were another important factor of the tourism development of the Basin, especially for non-provincial capital city. No matter the domestic tourism or inbound tourism, the cities with better tourism all had rich and high-profile cultural attractions. For example, Jinzhong city was famous for the Ancient City of Ping Yao, which was world culture heritage and attracted many tourists every year. Datong city and Luoyang city were both famous for the Buddhist culture, and the Yungang grottoes and Longmen grottoes were both world culture heritage. These cultural tourism resources greatly enhanced the popularity of the two cities. Another example is mountain Tai, which was known as the stone carving of ancient kings in China, and lead the tourism development of Tai’an city to catch up with the provincial capital.

The interregional relationship is another factor affecting the spatial patterns of tourism economy in the Yellow River Basin. There are many large-scale geographical entities in the interregional areas covering multiple prefectures in the basin. However, the interregional tourism developed relative independently of the neighboring prefectures due to the administrative divisions. And the lack of interregional cooperation could cause unbalanced utilization of the trans-boundary resources and environmental degradation on both sides of the boundary line [28]. For example, Yuntai Mountain is located at the junction areas of Jiaozuo in Henan and Jincheng in Shanxi. The part in Henan Province is famous for the title of 5A Scenic Spot, National Geological Park and National Scenic Spot, while the part in Shanxi Province is an ordinary tourist attraction with less popularity and tourism economic benefits. The 5A Scenic Spot in Henan brings more economic income than the ordinary tourism attraction in Shanxi Province.

Last but not the least, the infrastructure condition is a crucial factor on the tourism development in the Yellow River Basin. The regional accommodation and transportation facilities are essential and supportive factors for tourism development. With better public facilities, the tourism resources could attract more visitors and earn more income. For example, the construction of highway, railway, airline and tourists’ reception facilities in Yan’ an city improved significantly during the study period, and the total investment in fixed assets of the city increased by 6.9% from 2017 to 2018. In 2018, the city attracted 63.45 million tourists and earned 5.88 billion dollars, which were 1.25 times and 1.38 times more than that in 2017.

## Conclusion

Based on the tourism economic data of 73 prefectural units of the Yellow River Basin, this study analyzed the spatial pattern of tourism economy (including tourists’ number and tourism income) and the influencing factors in the Basin. The numerical features, spatial variation, and spatial association of the tourism economy at prefectural scales in the basin were acquired. The effects of different factors on the tourism development were discussed from the aspects of the location, the terrain, the cultural resources, the interregional relationship and the infrastructure. The results of the study are summarized as follows:

(1) At provincial level, Shanxi Province had the largest number of tourists and tourism income, while Ningxia had the smallest number of tourists and the least tourism income. The prefectures in Shaanxi Province had the largest number of the average tourists’ number and highest average tourism income, while the prefectures in Ningxia had the least number of tourists and lowest average tourism income. At prefectural level, Xi’an city hosted the most tourists and received the highest tourism income, while Golog Tibetan Autonomous Prefecture had the smallest number of tourists and the lowest tourism income. In addition, the fastest growth prefectural unit was Alxa League, and the slowest growth prefectural unit was Golog Tibetan Autonomous Prefecture. Compared with the inbound tourism, the development of domestic tourism in the Yellow River Basin is much more imbalanced.

(2) The spatial variance of tourism income is larger than the tourists’ number, indicating the spatial pattern of the tourism income was more unbalanced than that of tourists’ number. There were significant spatial associations in tourists’ number and tourism income. Overall, the significant high-high clusters and low-low clusters were gathered in the east and west of the Yellow River Basin, respectively. The central and eastern prefectures are the hot spots of tourism economy in Yellow River Basin, while the western prefectures are the cold spots.

(3) The spatial patterns of tourism economy in the Yellow River Basin is influenced by many factors including location, terrain, culture resources, regional policies, the interregional relationships and infrastructure. The tourism economy had a negative relationship with the distance between to capital cities. The largest tourism economy occurred where the relative elevation was between 1500-2000m, and the tourism flow and income decreased when the relative elevation was higher than 2000m. Cities with more cultural tourism resource usually attract more tourists and resulted with more income. The administrative divisions resulted different development of the same tourism resources. The investment in tourism infrastructure could stimulate the tourism economy significantly.

The study will be helpful for the tourism management in the Yellow River Basin or other interprovincial areas. Due to the limit of the data, we only analyzed the influencing factors of the spatial pattern of tourism economy by qualitatively analysis. Future studies can use econometric model with panel data, which will be more practical for describing the differences in tourism development.
